# Fundamental and Targeted Approaches in Pulmonary Arterial Hypertension Treatment

**DOI:** 10.3390/pharmaceutics17020224

**Published:** 2025-02-10

**Authors:** Ji Su Park, Yong Hwan Choi, Ji-Young Min, Jaeseong Lee, Gayong Shim

**Affiliations:** 1 School of Systems Biomedical Science, Soongsil University, Seoul 06978, Republic of Korea; jisu6042@soongsil.ac.kr (J.S.P.); iphone20011@soongsil.ac.kr (Y.H.C.); jymin0305@soongsil.ac.kr (J.-Y.M.); jsg@soongsil.ac.kr (J.L.); 2 Integrative Institute of Basic Sciences, Soongsil University, Seoul 06978, Republic of Korea

**Keywords:** pulmonary arterial hypertension, fundamental therapy, targeted therapy, nanoparticle-based drug delivery systems, pulmonary delivery systems

## Abstract

Pulmonary arterial hypertension (PAH) is a chronic and progressive disease marked by vascular remodeling, inflammation, and smooth muscle cell proliferation, with limited treatment options focused primarily on symptom management. The multifactorial nature of PAH, encompassing genetic, autoimmune, and connective tissue contributions, complicates its treatment, while irreversible vascular changes, such as fibrosis, remain unaddressed by current therapies. Fundamental research on molecular pathways and targeted delivery systems has paved the way for advanced therapeutic strategies that aim to modify disease progression rather than merely manage symptoms. Nanoparticle-based drug delivery systems, leveraging controlled release and pulmonary targeting, offer a promising avenue to overcome these challenges. Such systems enable precise localization to pulmonary vasculature, minimize systemic side effects, and support emerging approaches like gene therapy and combination treatments. Future research should focus on refining nanoparticle formulations for personalized medicine, optimizing inhalation delivery systems, and integrating multi-target approaches to achieve curative outcomes in PAH. This review explores pathophysiology of PAH, current pharmacological strategies, and innovative nanoparticle-based therapies, emphasizing their potential to transform PAH treatment and address its underlying mechanisms.

## 1. Introduction

Pulmonary arterial hypertension (PAH) is a chronic and progressive disease characterized by elevated pulmonary arterial pressure due to vascular remodeling, inflammation, and smooth muscle cell proliferation [1,2]. Despite advancements in understanding the molecular and cellular mechanisms of PAH, current therapies focus primarily on symptom relief and slowing disease progression, failing to provide a definitive cure. PAH arises from a combination of factors, including autoimmune disorders, genetic predispositions, and connective tissue diseases, which makes it challenging for any single therapy to address the root causes. Furthermore, the disease is marked by irreversible structural changes in the pulmonary vasculature, such as vascular remodeling, thickening, and fibrosis, which current pharmacological options cannot reverse. This multifactorial pathophysiology and the limited scope of available treatments underscore the significant clinical challenges posed by PAH.

Fundamental treatment of PAH requires innovative therapeutic options, including gene therapies. Nucleic acid-based drugs, such as siRNA, miRNA, or mRNA, hold promise for correcting genetic abnormalities underlying the disease. However, these therapies are inherently unstable, with short half-lives, necessitating targeted delivery systems to ensure efficacy [3]. Additionally, PAH often arises from diverse pathological factors, requiring the simultaneous targeting of multiple mechanisms. Combination therapies, leveraging either multi-drug loading on a single nanoparticle platform or the concurrent use of multiple nanoparticle systems, provide a versatile strategy to address these complex etiologies effectively [4].

For the successful application of nanomedicine in PAH treatment, efficient pulmonary drug delivery is paramount. The lungs, with their extensive surface area and rich blood supply, represent an ideal route for drug administration. However, challenges such as inadequate drug accumulation in target sites or rapid systemic absorption often lead to suboptimal therapeutic outcomes and systemic side effects. Non-invasive approaches like inhalation or intratracheal injection allow for localized drug action while minimizing systemic exposure. Pulmonary-targeted strategies employing nanoparticles can enhance drug stability, control release kinetics, and enable selective accumulation at the target site, overcoming many limitations of current PAH therapies.

**Figure 1:**
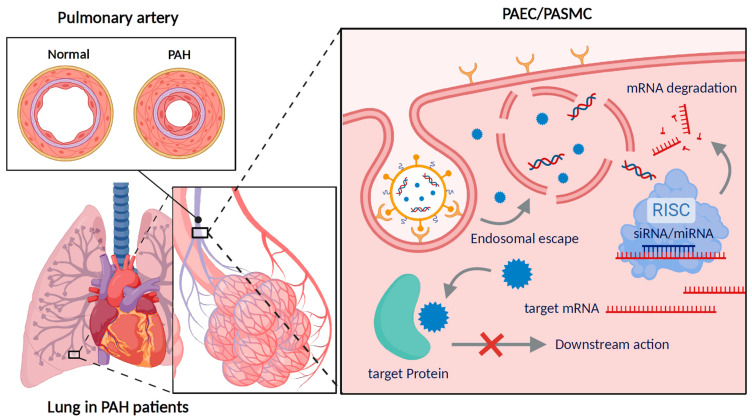
Schematic representation of a nanocarrier-based approach targeting the fundamental causes of PAH. The diagram illustrates the pathological changes in the pulmonary artery of PAH patients and highlights the mechanism of nanocarrier-mediated delivery to pulmonary arterial endothelial cells and pulmonary arterial smooth muscle cells. Created in BioRender. Jisu, P. (2025) https://BioRender.com/y93a096, accessed on 30 December 2024.

This review explores the pathophysiology of PAH, the pharmacological mechanisms of existing treatments, and the potential of nanoparticle-based drug delivery systems to revolutionize PAH management (Figure 1). Furthermore, it highlights pulmonary-targeted drug delivery strategies and innovative therapeutic developments, providing insights into a new paradigm for PAH treatment.

## 2. Pathophysiology of PAH

Pulmonary hypertension (PH) is characterized by a mean pulmonary arterial pressure of at least 25 mmHg, primarily due to obstructive changes in the pulmonary vasculature [5,6,7,8]. PH is classified into five groups based on pathophysiological characteristics. Group 1, PAH, includes idiopathic and heritable forms and is often linked to connective tissue diseases and congenital heart defects [9]. Group 2 results from left heart disease, such as left ventricular dysfunction, and valvular diseases like mitral valve disease [10]. Group 3 is associated with lung diseases or hypoxia, commonly due to chronic obstructive pulmonary disease or interstitial lung disease [11]. Group 4 involves pulmonary artery obstructions, primarily caused by chronic thromboembolic pulmonary disease [12]. Group 5 includes cases with unclear or multifactorial mechanisms [13]. PAH can develop due to various etiologies and typically progresses with symptoms such as dyspnea, fatigue, and right heart failure. Diagnosis involves transthoracic echocardiography and is confirmed through right heart catheterization [9]. Delayed treatment increases mortality risk due to progressive pulmonary vascular remodeling and right ventricular failure.

The progression of PAH involves pulmonary vascular remodeling, characterized by intimal thickening, fibrosis, thrombotic lesions, and inflammatory cell infiltration. Key cellular contributors include pulmonary arterial endothelial cells and smooth muscle cells, which respond to inflammatory stimuli and oxidative stress, exacerbating disease progression [14]. Genetic and environmental factors such as hypoxia, infections, and air pollutants contribute to the disease by promoting endothelial cell damage and vascular remodeling [15,16]. Genetic mutations in TGF-β superfamily proteins, such as BMPR2, ALK1, and ENG, play a significant role in heritable PAH by disrupting pulmonary vascular homeostasis [17,18]. Additional mutations, including KCNK3 and ATP13A3, contribute to vascular stiffening and endothelial dysfunction [19,20]. Chronic inflammation is another key factor driving PAH progression. Inflammatory cytokines, such as IL-6 and IL-1β, promote endothelial and smooth muscle cell proliferation and migration, contributing to vascular remodeling [21]. Autoimmune diseases and genetic predispositions further exacerbate inflammatory responses, accelerating disease progression through increased cytokine expression and mitochondrial dysfunction [22]. Understanding the interplay of these factors is essential for developing targeted therapeutic strategies to manage PAH effectively.

## 3. Drug Classes and Mechanisms in PAH

The therapeutic goals for PAH include inducing vasodilation, suppressing inflammation and cellular proliferation, and alleviating vascular remodeling [23]. Current pharmacological treatments for PAH primarily focus on managing symptoms, reducing pulmonary arterial pressure, and slowing disease progression rather than offering a definitive cure (Table 1). These therapies address the complex pathology of the disease by targeting key mechanisms associated with vascular dysfunction.

### 3.1. Conventional Approaches for PAH Management

PAH treatments currently used in clinical practice can be categorized into four main types: (i) endothelin receptor antagonists, (ii) phosphodiesterase type 5 inhibitors, (iii) drugs targeting the prostacyclin pathway, and (iv) soluble guanylate cyclase activators [24,25,26].

#### 3.1.1. Endothelin Receptor Antagonists

Endothelin acts as a potent blood vessel constrictor in lung tissue and is elevated in patients with PAH [27]. Endothelin functions as both a pulmonary vasoconstrictor and a smooth muscle mitogen, leading to a reduction in pulmonary arterial lumen diameter and an increase in pulmonary arterial pressure, which can contribute to the development of PAH [28]. Endothelin receptor antagonists block endothelin receptors, thereby inhibiting the progression of PAH caused by elevated endothelin levels. These antagonists are classified into two types: nonselective receptor antagonists (e.g., bosentan, macitentan), which block both endothelin A (ETA) and endothelin B (ETB) receptors, and selective receptor antagonists (e.g., ambrisentan), which block only the ETA receptor [29,30]. In PAH animal models, an imbalance in the expression of ETA and ETB receptors has been observed, with an initial upregulation of ETA receptors and a subsequent increase in ETB receptor expression in PASMCs, both of which have been implicated in PAH progression.

#### 3.1.2. Phosphodiesterase Type 5 Inhibitors

Phosphodiesterase type 5 (PDE5) degrades cyclic guanosine monophosphate (cGMP), thereby inhibiting the nitric oxide (NO)-mediated relaxation of smooth muscle and contributing to the development of PAH [31,32,33,34]. NO activates guanylate cyclase, increasing cGMP production, which promotes vasodilation of smooth muscle by upregulating K^+^ channels, inhibiting Ca2^+^ channels, and reducing intracellular Ca2^+^ levels. In the PASMCs of PAH patients, PDE5 expression is upregulated, leading to enhanced cGMP degradation and excessive PASMC contraction. PDE5 inhibitors increase cGMP and NO levels, inducing PASMC relaxation and offering an effective therapeutic approach for PAH.

Sildenafil, an FDA-approved competitive inhibitor of cGMP-specific PDE5, enhances NO signaling, reducing pulmonary arterial pressure and promoting vasodilation, thereby alleviating PAH symptoms [35]. Tadalafil, another FDA-approved PDE5 inhibitor for PAH treatment, effectively regulates smooth muscle contraction and promotes vasodilation in the pulmonary vasculature [36]. Vardenafil, primarily used for erectile dysfunction, has demonstrated greater efficacy in PDE5 inhibition compared to sildenafil and tadalafil [37,38]. In monocrotaline (MCT)-induced PAH rat models, vardenafil reduced pulmonary artery pressure, alleviated vascular remodeling and right ventricular (RV) hypertrophy, inhibited PASMC proliferation, and mitigated oxidative stress [37].

#### 3.1.3. Prostacyclin Pathway-Targeted Drugs

Prostacyclin (prostaglandin I2, PGI2) is synthesized in vascular endothelial cells and suppresses Ca^2^⁺ influx, inducing vasodilation and inhibiting smooth muscle cell proliferation and platelet aggregation [39]. Reduced prostacyclin levels lead to pulmonary endothelial dysfunction, a key contributor to PAH [40,41]. Beraprost, an orally administered prostacyclin analog, targets prostacyclin (IP) and prostaglandin E2 receptor subtype 3 (EP3). Studies in monocrotaline (MCT)-induced PAH rat models have demonstrated that beraprost effectively reduces pulmonary arterial remodeling and right ventricular (RV) hypertrophy [42]. Iloprost, an FDA-approved inhaled prostacyclin analog, activates IP receptors and inhibits EP1, EP3, and EP4 activation, preventing vasoconstriction [43]. Treprostinil palmitil, a prodrug designed for sustained release, overcomes the short half-life of treprostinil and exhibits enhanced efficacy in alleviating pulmonary vasoconstriction [44].

#### 3.1.4. Soluble Guanylate Cyclase Activators

Soluble guanylate cyclase (sGC) is a key enzyme in the nitric oxide (NO)-cGMP signaling pathway that mediates smooth muscle relaxation and vasodilation. In PAH, endothelial dysfunction results in reduced NO bioavailability, leading to impaired sGC activation and decreased cGMP levels, which contribute to increased pulmonary arterial pressure and vascular remodeling [45]. sGC activators, such as riociguat, directly stimulate sGC independently of NO, enhancing cGMP production and promoting vasodilation in pulmonary arteries. This mechanism bypasses NO deficiency and restores vascular homeostasis. Riociguat, an FDA-approved sGC activator, has demonstrated efficacy in reducing pulmonary arterial pressure and improving exercise capacity in PAH patients by enhancing endothelial function and reducing right ventricular afterload [46].

### 3.2. Current Pharmacological Approaches

Rho kinase inhibitors, such as fasudil, have emerged as promising therapeutic agents by targeting key pathological processes, including vascular contraction, PASMC proliferation, and endothelial dysfunction [47]. Rho kinase plays a pivotal role in actin cytoskeleton organization and vasoconstriction, and its overactivation contributes to the increased vascular tone observed in PAH. Fasudil, delivered via liposomal inhalation, demonstrated sustained reductions in pulmonary arterial pressure in monocrotaline (MCT)-induced PAH rat models, effectively alleviating vascular remodeling without causing systemic vasodilation, thus minimizing adverse effects [48].

Inflammation and oxidative stress are also critical contributors to PAH pathogenesis, as chronic inflammatory responses exacerbate endothelial injury and promote abnormal PASMC proliferation [49]. Reactive oxygen species (ROS) generated in PAH patients impair endothelial cell function and enhance PASMC proliferation, accelerating vascular remodeling and disease progression.

To counteract oxidative stress, epigallocatechin gallate (EGCG), a natural antioxidant and anti-inflammatory agent, has shown potential in inhibiting TGF-β signaling pathways, thereby reducing PASMC proliferation and fibrosis [50]. However, its clinical utility is limited by low stability and a short plasma half-life, prompting the development of liposomal inhalable formulations to enhance drug bioavailability and pulmonary retention.

Another promising antioxidant approach is ethyl pyruvate, which functions as both a ROS scavenger and a high-mobility group box 1 (HMGB1) inhibitor. Ethyl pyruvate has demonstrated attenuation of PAH progression in preclinical models by reducing inflammatory cytokine release and oxidative damage in pulmonary vasculature [51].

Combination therapies, which integrate drugs with distinct mechanisms of action, are gaining attention for their synergistic effects in PAH treatment. For example, the co-administration of sildenafil and simvastatin has been shown to synergistically inhibit PASMC proliferation and vascular remodeling, offering an improved therapeutic outcome compared to monotherapy [52]. Similarly, the combination of fasudil with superoxide dismutase (SOD) effectively mitigated PAH progression and right ventricular hypertrophy in preclinical models by targeting multiple pathological pathways concurrently [47].

## 4. Targeted Strategies in PAH Treatment

Despite these advances, PAH treatments remain limited in their capacity to offer a cure, largely due to the complex and multifactorial nature of the disease. Research into fundamental treatments for PAH is ongoing, with emerging strategies focusing on direct targeting the mechanisms driving vascular remodeling and repair damaged vasculature. Promising agents such as sotatercept have shown potential in modulating vascular remodeling through pathways like bone morphogenetic protein (BMP) signaling, offering hope for more effective intervention. However, these therapies are still considered adjunctive rather than curative, emphasizing the need for continued innovation in PAH treatment. Current PAH therapies face significant limitations, including drug instability, short duration of action, nonspecific distribution, and formulation constraints. Moreover, many drugs effective against PAH exert systemic effects on the circulatory system, leading to dose limitations and heightened risks of adverse effects. These shortcomings underscore the need for advanced therapeutic strategies that can address the disease more precisely and effectively.

To overcome these obstacles, the development of nanoparticle-based drug delivery systems incorporating controlled-release technologies and targeted delivery strategies has garnered significant attention. PAH is considered a suitable candidate for such approaches due to its localized pathology within the pulmonary vasculature. Nanoparticles, composed of either naturally occurring or synthetically engineered materials, offer the ability to modulate drug release kinetics and preferentially accumulate drugs at target sites, thereby enhancing therapeutic efficacy while minimizing systemic side effects. This dual approach—combining fundamental treatment development with pulmonary-targeted delivery—represents a promising avenue for addressing the limitations of current PAH therapies and advancing toward curative solutions.

### 4.1. Endothelin Receptor Antagonists

Bosentan (Bos) is a dual endothelin receptor antagonist (ETA and ETB) developed specifically for PAH treatment. Bosentan is poorly water-soluble, with its solubility highly dependent on pH [53]. Drugs with solubility below 1 mg/mL at physiological pH often exhibit bioavailability issues [42]. To address this, self-nanoemulsifying drug delivery systems (SNEDDS), which are isotropic mixtures of oil, surfactants, and co-surfactants, have been explored to improve Bosentan solubility and bioavailability [53]. Compared to commercial formulations like Tracleer^®^, SNEDDS-loaded Bosentan demonstrated 3- to 8-fold higher dissolution rates in physiological media such as FaSSIF and FeSSIF. Another approach involves developing nanocomposites using Soluplus^®^ polymers through single-emulsification and freeze-drying methods [29]. These nanocomposites, with particle sizes below 100 nm, exhibited reduced crystallinity and improved amorphous stability over six months. Such strategies enhance bosentan solubility and bioavailability, offering a promising platform for PAH therapy.

### 4.2. PDE5 Inhibitors

Sildenafil (SD), a selective PDE5 inhibitor, prevents pulmonary arterial remodeling but suffers from poor pulmonary accumulation, a short half-life, and systemic side effects. To overcome these limitations, glucuronic acid (GlcA)-modified liposomes were developed to deliver SD specifically to PASMCs [54]. These liposomes showed a 32.4% reduction in pulmonary arterial pressure (PAP), a 40% decrease in medial thickness, and a 45% improvement in right ventricular hypertrophy compared to free SD. Additionally, SD inhalation formulations have been investigated, including co-delivery systems using poly(lactic-co-glycolic acid) (PLGA) polymers [54,55]. In preclinical models, inhalable SD and rosiglitazone-loaded PLGA particles reduced mean PAP, mitigated pulmonary artery remodeling, and improved RV hypertrophy [56]. Although the chronic safety of combination therapies needs further validation, these formulations offer a viable alternative for PAH treatment.

### 4.3. Prostacyclin Pathway-Targeted Drugs

Prostacyclin (PGI2) synthesis is impaired in PAH patients, contributing to vascular remodeling [57]. While epoprostenol sodium infusion offers therapeutic benefits, it is associated with severe side effects like hypotension and catheter-related infections due to its short half-life. To address these issues, PLGA nanoparticles loaded with beraprost sodium have been developed, showing anti-proliferative and pro-apoptotic effects in PAH models [58]. Liposomal inhaled iloprost has shown potent vasorelaxation effects in U-46619-induced PAH rat models [43]. Treprostinil binds to IP and EP2 receptors, suppressing transforming growth factor-β1 (TGF-β1) and connective tissue growth factor expression, thereby reducing PASMC proliferation and migration [59]. Treprostinil palmitil liposomal inhalation aerosols have also demonstrated extended pharmacokinetic profiles, with over 12 h of sustained activity, making them suitable for once-daily inhalation. Additionally, GlcA-modified liposomes loaded with treprostinil target GLUT-1 overexpression in PASMCs, leading to superior PAP reduction and sustained pulmonary accumulation [59]. These innovations in prostacyclin delivery provide targeted and efficient PAH management.

### 4.4. Other Strategies

Fasudil, a rho kinase inhibitor, is formulated using PEGylated PLGA liposomes for inhalation therapy [48]. These nanoparticles have reduced pulmonary arterial remodeling and sustained vascular dilation in PAH models. CAR-conjugated liposomes, targeting heparan sulfate, have also been developed to enhance specific pulmonary delivery, reducing off-target effects [60]. CAR liposomes co-encapsulating fasudil and superoxide dismutase demonstrate prolonged drug release and improved anti-PAH efficacy [61]. Additionally, bioinspired nanoerythrosome (NER)-based formulations have been used to encapsulate fasudil [62]. Compared to intravenous bolus injections, CAR–NER–fasudil extended vasodilation effects from 60–80 min to over 200 min and achieved a 1.5-fold greater reduction in mean PAP. These advanced formulations underscore the potential of nanotechnology in optimizing PAH therapies.

## 5. Pulmonary Targeting of Nanoparticle Systems for Disease Treatment

Pulmonary drug delivery is a form of drug targeting where therapeutic agents are delivered to the active site in the lungs for localized action or to absorption sites for systemic effects [63,64]. The former approach offers advantages such as relatively low doses, a low incidence of systemic side effects, and a rapid onset of action. Additionally, molecules designed to achieve systemic effects can also be delivered via the pulmonary route, circumventing low absorption rates through the gastrointestinal (GI) tract and avoiding the need for injectable formulations. The pulmonary epithelium, with its vast surface area of approximately 100 m^2^ and its rich blood supply, is increasingly recognized as a promising non-invasive route for both localized and systemic drug delivery [65]. This pathway is applicable not only to treating conditions like asthma, localized infections, and pulmonary hypertension but also for delivering systemic therapeutics such as insulin, human growth hormone, and oxytocin.

As important as drug mechanisms and development is the design of effective targeting systems. Precisely delivering drugs to specific organs minimizes undesired toxicity and maximizes therapeutic efficacy. The lung, as a respiratory organ, connects to the trachea via bronchi and is anatomically divided into the conducting zone (trachea, bronchi, and bronchioles) that transports air and the respiratory zone (alveoli and airways) responsible for gas exchange [66]. The airway epithelium comprises the progressively thinning columnar epithelium, with the bronchial and bronchiolar layers measuring approximately 3.5 mm and 0.5–1 mm in thickness, respectively. The lung contains over 300 million alveoli composed of type I and type II alveolar cells interspersed with an extensive capillary network [67]. As oxygen moves from the alveoli to the bloodstream, it traverses a respiratory membrane approximately 0.5–1.0 μm thick, consisting of alveolar and capillary walls. This structure also facilitates drug absorption. The high permeability of the respiratory membrane, extensive surface area, and sufficient blood flow prevent first-pass metabolism and enable drug accumulation in the lungs, making it ideal for treating pulmonary diseases [68].

**Figure 2:**
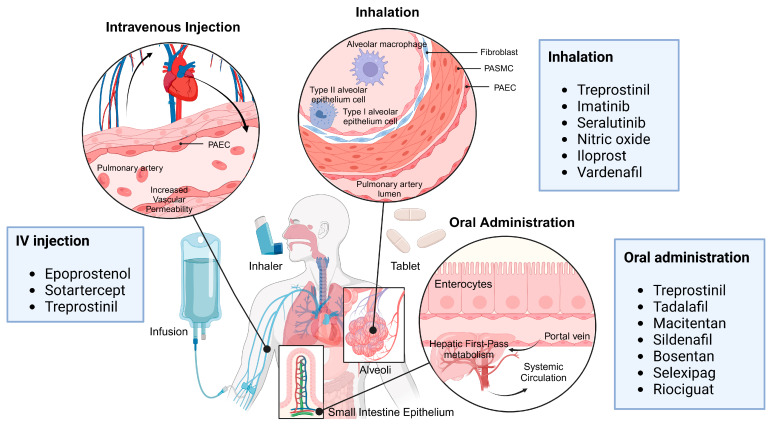
Routes of Pulmonary Drug Delivery. Illustration of pulmonary drug delivery methods, including nebulization, dry powder inhalers (DPI) or metered dose inhalers (MDI), and infusion. These delivery routes highlight the variety of strategies employed for effective pulmonary administration. Created in BioRender. Jisu, P. (2025) https://BioRender.com/y93a096.

Therapeutic strategies for PAH using nanocarrier systems, where the majority of approaches employ intratracheal administration routes for enhanced efficacy, are summarized in Table 2. In clinical settings, intratracheal injection is primarily utilized in preclinical animal studies and is rarely employed in human trials due to its inherent limitations. Instead, less invasive alternatives, such as inhalation delivery via nebulizers or inhalers, are widely adopted for pulmonary drug delivery, offering enhanced patient safety and convenience (Figure 2). In specific cases, intubation-assisted drug administration may be used, particularly in critical care settings, but its application remains limited. For intratracheal injection to gain broader clinical applicability, technological advancements ensuring minimal invasiveness and improved safety, such as nanocarrier-based drug formulations or precision catheter systems, would be essential.

Although pulmonary delivery has attracted considerable scientific interest over the past few decades, several challenges remain. For instance, therapeutic effects may dissipate quickly, necessitating frequent dosing (e.g., three to four times daily), and some drugs, such as bronchodilators, are rapidly absorbed through the pulmonary epithelium, leading to systemic side effects. However, recent advances in respiratory drug delivery strategies have led to significant improvements in delivery methods and devices. These advancements form the foundation for designing effective drug delivery systems tailored to pulmonary disease treatment.

### 5.1. Pulmonary Drug Delivery Route

#### 5.1.1. Inhalation

Inhalation is a non-invasive method of delivering drugs directly to the airway, maximizing therapeutic effects through high bronchial deposition and effective targeting [94]. The success of inhalation therapy depends heavily on particle size, stability, and the choice of an appropriate inhaler, requiring formulations with optimized physicochemical properties [95]. For effective inhalation, particle size must be ≤5 μm to ensure deposition in the bronchioles and alveoli, with an aerodynamic size ≤5 μm and high fine particle fraction (FPF), which considers particle density and shape for optimal lung deposition. In pulmonary arterial hypertension (PAH) treatments targeting the alveolar area, particles should be designed to be ≤3 μm. Particles smaller than 1 μm can avoid mucociliary and alveolar macrophage clearance, enabling effective pulmonary deposition. Additionally, larger particles (>5 μm) pose a risk of deposition in the upper respiratory tract and potential systemic circulation through absorption, which raises safety concerns when administered intravenously.

Inhaled liposomes are preferred for pulmonary delivery due to their phospholipid composition, which mimics lung surfactants, allowing for effective drug localization in the lungs [96]. They enable sustained drug release, improve stability and permeability, reduce systemic side effects, and enhance patient compliance. Studies and clinical trials on inhaled liposome formulations for treating various pulmonary diseases have been conducted [97]. Although inhalable liposomal arikace for mycobacterial infection has received FDA approval, nanomedicine-based inhaled formulations for PAH treatment remain at the preclinical research stage.

Inhalation therapy employs three primary inhaler types: nebulizers, dry powder inhalers (DPIs), and pressurized metered-dose inhalers (pMDIs) [98]. Nebulizers generate liquid aerosols without the need for propellants, enabling uniform drug delivery to deep lung tissues via respiration, making them a preferred dosage form [99]. Research on liposome-based formulations delivered through nebulization has demonstrated high FPF (53.46%) and suitable MMAD (4.41 μm), enabling effective drug deposition in the alveolar region of PAH lungs [50]. Vibrating mesh nebulization techniques minimize lipid bilayer damage, reducing drug loss and aggregation while improving delivery efficiency. Similar to liposomes, solid lipid nanoparticles (SLNs) or biodegradable and biocompatible PLGA (poly(lactic-co-glycolic acid)) nanoparticles can form respirable aerosols [100]. These nanoparticles minimize lung tissue damage and enable effective pulmonary delivery via nebulizers. PLGA nanoparticles, with their porous structure and low density, allow for sustained drug release and efficient lung deposition [74]. For drugs prone to aggregation and toxicity at high concentrations, nebulization provides uniform and targeted lung delivery, reducing toxicity and enhancing therapeutic outcomes [101]. pMDIs use propellants to deliver aerosolized drugs to the lungs effectively [94]. Liposome-based pMDI formulations enable sustained drug release, reduced systemic exposure, and improved therapeutic outcomes. Aerosolized liposome formulations with MMAD (1.54 μm) and FPF (74%) demonstrated effective lung targeting, reduced cough reflex, and improved patient compliance. DPIs deliver nanomedicine-based powders formulated using spray-drying techniques, ensuring drug stability and direct deposition into deep lung tissues [99]. Formulations using excipients like mannitol exhibit respirable particle sizes (1–5 μm) and high FPF, enabling sustained drug release and effective lung deposition [102]. For hydrophobic drugs (e.g., simvastatin), DPIs are particularly useful, as the drugs dissolve in the lungs for immediate therapeutic effects [31]. These formulations are easy to store and use without refrigeration or solubilization, although they pose challenges in dosage accuracy, drug wastage, and nasal cavity deposition. A dry powder formulation developed to enhance bioavailability and reduce systemic side effects showed a 13.7-fold increase in solubility compared to free tadalafil [103]. Intratracheal insufflation demonstrated significant improvements in MRT (2.3-fold) and tmax (3.7-fold) compared to oral administration. Widely used as a bronchodilator for asthma treatment, SS encapsulated in liposome carriers and delivered via DPI exhibited sustained drug release (90% over 14 h) [104].

#### 5.1.2. Intratracheal Injection

Intratracheal (IT) injection delivers drugs directly to the lungs with high precision and efficiency, requiring only small amounts. It minimizes nasal cavity deposition and systemic distribution, reducing side effects while ensuring effective lung deposition. IT injection is recognized as an effective delivery route for PAH and other pulmonary diseases. Administering aerosolized drugs via IT injection ensures uniform distribution and effective deposition in deep lung areas, maximizing therapeutic outcomes.

Studies using polymer-based aerosol nanoparticles have demonstrated successful drug delivery to lung tissues in PAH animal models, alleviating symptoms and slowing disease progression [79]. Liposome and lipid nanoparticle (LNP)-based aerosols have improved bioavailability and sustained drug release, achieving high therapeutic efficacy through IT injection [48,105]. Ligand-modified nanoparticles designed for cell-specific delivery have also been extensively studied [60,73]. For example, CAR-modified liposomes target overexpressed heparan sulfate in PASMCs, selectively accumulating in proliferative SMCs of PAH lungs [60]. When delivered via aerosolized IT injection, systemic entry was reduced and cellular uptake increased, enhancing therapeutic effects. While IT injection offers high delivery accuracy and low drug loss, it carries risks of lung tissue damage from repeated administration and requires anesthesia, limiting its use in some scenarios. Nevertheless, IT injection remains a promising strategy for pulmonary drug delivery.

### 5.2. Non-Pulmonary Drug Delivery Route

#### 5.2.1. Intravenous Injection

Intravenous (IV) injection is a common method for administering large volumes of drugs, avoiding first-pass metabolism and providing the potential for delivering drugs to the lungs [106]. IV-administered particulate drug delivery systems are designed to selectively lodge in pulmonary capillaries, requiring particle sizes slightly larger than the capillary diameters, which are approximately 7.5 ± 2.3 μm in healthy adults and 6.6 ± 1.6 μm or 7.5 ± 1.7 μm in rats and dogs, respectively. Many drugs are encapsulated in carriers such as microspheres, microcapsules, liposomes, and nanoparticles for delivery to the lungs via IV routes. These carriers regulate drug distribution and release, prolong drug action, and improve therapeutic efficacy and patient compliance. IV injection leverages increased vascular permeability in PAH to efficiently accumulate nanoparticles using passive targeting mechanisms. This approach mitigates the limitations of systemic drug distribution and enables lung-specific delivery through surface modifications of nanoparticles.

IV drug delivery strategies include the use of ligand-modified nanoparticles to selectively deliver drugs to lung tissues [59]. Glucuronic acid (GlcA), a glucose analog, targets glucose transporter-1 (GLUT-1), which is overexpressed in PAH PASMCs, enhancing pulmonary drug distribution and cellular uptake. Such approaches have demonstrated selective accumulation of nanoparticles in pulmonary arteries and effective inhibition of vascular remodeling [107]. Cell-penetrating peptides like octaarginine (R8) have been used to enhance lung accumulation of liposomes. Similarly, nanoparticles conjugated with E-selectin ligands exhibit high specificity for pulmonary vascular endothelium, achieving efficient delivery [108]. Additionally, surface modifications with polymers such as polyethylene glycol (PEG) and polyethylenimine (PEI) improve lung targeting [109]. PEG reduces opsonization and serum protein interactions, extending blood circulation time and enhancing nanoparticle stability. PEI provides a positive charge to nanoparticles, facilitating their interaction with the negatively charged lung endothelial glycocalyx and promoting cellular uptake.

#### 5.2.2. Intraperitoneal Injection

Intraperitoneal (IP) injection delivers drugs into systemic circulation, maintaining consistent drug concentrations and serving as an effective method for poorly soluble drugs [76]. Emulsion-based drugs administered via IP injection have been shown to reduce migration and proliferation of PAH PASMCs [110]. However, IP injection has been associated with systemic side effects, such as decreased systolic blood pressure and liver damage, making it less commonly used for PAH treatment [76]. Research targeting lung tumors has utilized HA-PEI nanoparticles to deliver miR-125b to peritoneal macrophages [111]. HA selectively binds to macrophages and directs them to inflammation sites, successfully targeting tumor-associated macrophages (TAMs) and achieving significant outcomes in cancer immunotherapy.

#### 5.2.3. Oral Administration

Oral administration is widely preferred due to its simplicity and high patient compliance [53,112]. However, it is limited by the gastrointestinal (GI) environment, which reduces drug stability, absorption rates, and bioactivity through first-pass metabolism [53]. PAH treatments include oral drugs such as bosentan, tadalafil, ambrisentan, and sildenafil. While oral formulations offer longer plasma half-lives than subcutaneous, intravenous, or inhaled forms, they present challenges in managing adverse effects such as hypotension or pulmonary edema [113].

Nanoparticle-based formulations have been developed to address these issues [112]. Solid lipid nanoparticles (SLNs) with an average size of 130 nm exhibit rapid initial drug release followed by sustained release patterns, improving drug stability and therapeutic efficacy. Lipid core nanocapsules (LNCs) extend drug half-lives, enhance bioavailability, and effectively suppress PAH progression [30]. Emulsion-based formulations further improve oral bioavailability and absorption of lipophilic drugs, such as ambrisentan, bosentan, and sildenafil, by bypassing first-pass metabolism and inhibiting P-glycoprotein-mediated drug efflux [35,53,114].

## 6. Conclusions

PAH remains a significant clinical challenge due to its complex pathophysiology and the limited efficacy of existing treatment options. Nanoparticle-based drug delivery systems for pulmonary targeting present a promising approach to address these challenges by offering precise drug localization, controlled release, and prolonged retention in the lungs, thereby minimizing systemic toxicity and enhancing patient compliance. Unlike conventional delivery methods that primarily focus on symptom management, nanoparticle-based strategies provide a means to directly interact with diseased pulmonary vasculature, facilitating targeted modulation of pathogenic pathways such as endothelial dysfunction, inflammation, and vascular remodeling.

Future research should focus on optimizing nanoparticle formulations in combination with advanced inhalation devices to ensure efficient pulmonary deposition and therapeutic efficacy. Additionally, the exploration of personalized medicine approaches, such as patient-specific nanoparticle formulations tailored to genetic profiles, may further enhance treatment outcomes. Ultimately, the integration of cutting-edge drug delivery technologies and molecular therapies offers a transformative opportunity to improve PAH management and enhance the quality of life for patients.

## Figures and Tables

**Table 1:** Approved drugs for PAH treatment.

Drug Class	Drug	Mode of Action
Endothelin receptor antagonists	Bosentan monohydrate	Reduces the pathogenic effects of elevated endothelin-1 in PAH
	Macitentan	Reduces vasoconstriction and smooth muscle cell proliferation in pulmonary arteries
	Ambrisentan	Inhibits vasoconstriction and cell proliferation while preserving ETB receptor-mediated vasodilation and ET-1 clearance
PDE5 inhibitor	Sildenafil citrate	Increases cGMP levels in pulmonary vascular smooth muscle cells, inducing vasodilation of the pulmonary vascular bed
	Tadalafil	Increases cGMP levels to enhance smooth muscle relaxation, reducing pulmonary arterial pressure and vascular remodeling in PAH
	Vardenafil hydrochloride	Increases cGMP levels to promote smooth muscle relaxation and vasodilation in the pulmonary vasculature in PAH
Prostacyclin analog	Epoprostenol sodium	Activates G protein-coupled receptors to increase cAMP, inhibiting platelet aggregation and inducing vasodilation
	Iloprost tromethamine	Mimics prostacyclin to induce vasodilation, reduce oxidative stress, and protect endothelial and mitochondrial function in PAH
	Treprostinil	Activates prostacyclin receptors to increase cAMP, inducing vasodilation, inhibiting platelet aggregation, and reducing inflammation in PAH
Selective IP receptor agonist	Selexipag	Promotes vasodilation, inhibits vascular smooth muscle proliferation, and reduces inflammation in PAH
Soluble guanylate cyclase stimulator	Riociguat	Increases cGMP generation to enhance vasodilation and reduce vascular remodeling in PAH
Activin receptor type IIA-Fc fusion protein	Sotatercept	Acts as a ligand trap for activin-class ligands to restore BMP signaling and reduce pulmonary vascular remodeling in PAH

**Table 2:** Nanocarrier-based pulmonary targeted strategies for PAH treatment.

Target	Therapeutic Cargo	Therapeutic Strategy	Core Materials for Carriers	Route	Animal Model	Ref.
BMPR2	SIN3a gene	Increase BMPR2 expression in the epigenetic pathway to overcome BMPR2 silencing caused by methylation	Adeno-associated virus serotype1 (AAV1)	Intratracheal (aerosol inhalation)	Sprague–Dawley rat	[69]
BOLA3	BOLA3 gene	Induce the expression of BOLA3, which plays a key role in PH pathogenesis	Lentiviral vector	Orotracheal instillation	C57BL/6 mice, Sprague–Dawley rat	[70]
Endothelin receptor	Ambrisentan	Prevent or reverse histological change caused by elevated levels of ET1, treatment of PAH	Chitosan	Intratracheal	n.a.	[71]
	Ambrisentan, NaNO2	Block the vasoconstrictive response in blood vessels, act as a pulmonary vasodilator	Perfluorooctyl bromide emulsion	Intratracheal	Sprague–Dawley rat	[72]
	Bosentan	Prevent or reverse histological change caused by elevated levels of ET1, treatment of PAH	PLGA	Intratracheal	Wistar Albino rat	[27]
Guanylate cyclase	Diethylenetriamine NONOate	Activate guanylate cyclase to produces vasodilation and reduce smooth muscle cell proliferation	Liposome (PEGylated)	Intratracheal	Rat	[73]
	Cinaciguat	Activate soluble guanylate cyclase (increase vasodilation of pulmonary arteries), decrease pulmonary arterial blood pressure	PLGA, polyvinyl pyrrolidone (PVP)	Intratracheal (dry powder insufflator)	Sprague–Dawley rat, Mini-Pig	[74]
HMG-CoA reductase	Cerivastatin	Inhibit smooth muscle cell proliferation, improve endothelial function, reduce inflammation and oxidative stress	Liposomes	Inhalation	Sprague–Dawley rat	[75]
IκB	H2S-releasing asprin derivative (ACS14)	Inhibit the EndMT process by suppressing IκB degradation and NF-κB activation	PLGA	Intratracheal	Sprague–Dawley rat	[76]
Pdgfb	Pdgfb siRNA	Prevent hypoxia-induced distal pulmonary arteriole muscularization, PH, and RVH	PPMS	Orotracheal instillation	C57BL/6 mice	[77]
Nuclear factor κB	Decoy oligonucleotide	Attenuate inflammation, proliferation, development of PAH and pulmonary arterial remodeling	PEG-PLGA	Intratracheal instillation	Rat	[78]
PDE5	Tadalafil	Increase level of cGMP and nitric oxide in pulmonary vasculature to reduce pulmonary arterial pressure	Nanoemulsion	Orotracheal instillation	Sprague–Dawley rat	[36]
	Sildenafil		PLGA	Intratracheal	Sprague–Dawley rat	[31]
			Carboxymethyl cellulose/sodium alginate hydrogel microparticle	Intratracheal	Albino rat	[79]
			PLGA	Intratracheal	Albino rat	[80]
PDE5, PPAR-γ	Sildenafil, Rosiglitazone	Increase level of cGMP and nitric oxide in pulmonary vasculature to reduce pulmonary arterial pressure. inhibit PASMC proliferation by modulating cell growth and apoptosis	PLGA	Intratracheal	Sprague–Dawley rat	[56]
PDGF-receptor tyrosine kinase	Imatinib	Reverse pulmonary vascular remodeling, anti-proliferative and pro-apoptotic effects	Liposomes	Intratracheal	Sprague–Dawley rat	[81]
PPAR-γ	Rosiglitazone	Inhibit PASMC proliferation by modulating cell growth and apoptosis	PLGA	Intratracheal	Sprague–Dawley rat	[82]
Prostaglandin E receptors	Prostaglandin E1	Vasodilatory, anti-inflammatory, anti-aggregatory, and anti-proliferative properties	PLGA	Intratracheal (aerosol inhalation)	Sprague–Dawley rat	[83]
			PLGA	Intratracheal	Sprague–Dawley rat	[84]
Rac1	miR-429-3p	Inhibit proliferation and migration of PASMCs, vascular remodeling of pulmonary arterial walls observed in PAH	Exosomes	Intratracheal	C57BL/6 mice	[85]
Rho-kinase	Fasudil	Dilate pulmonary arteries and arterioles, reduces arterial remodeling, prolonged pulmonary preferential vasodilation	Liposomes	Intratracheal	Sprague–Dawley rat	[48]
			Peptide–micelle hybrid particle	Intratracheal	Rat	[86]
			Nanoerythrosomes	Intratracheal	Sprague–Dawley rat	[87]
			Starch-coated magnetic liposomes (PEGylated)	Intratracheal	Sprague–Dawley rat	[88]
			Nanoerythrosomes	Intratracheal	Sprague–Dawley rat	[62]
			Liposome (PEGylated)	Intratracheal	Sprague–Dawley rat	[89]
			CAR-liposomes (PEGylated)	Intratracheal	Sprague–Dawley rat	[90]
			Liposome (PEGylated)	Intratracheal, intravenous	Sprague–Dawley rat	[60]
	Fasudil, SOD		Liposome (PEGylated)	Intratracheal	Sprague–Dawley rat	[61]
ROS, HMGB1	Ethyl pyruvate	Decreased levels of HMGB1, IL-6, TNFα, reactive oxygen species, and ET1 in lung improve pulmonary arterial remodeling	PEG-PLGA	Intratracheal	Sprague–Dawley rat	[51]
SIRT1	Resveratrol	Increased PASMC apoptosis to attenuate pulmonary arterial remodeling and the alleviation of PAH	Lipid nanoparticles	Intratracheal	Sprague–Dawley rat	[91]
TRPC1	TRPC1 siRNA	Attenuate PAH-associated RV and pulmonary arteriolar remodeling	Liposomes	Intratracheal	C57BL/6 mice	[92]
VEGFR2	VEGF, SDF	Block VEGF signaling to facilitate utilizing extra-pulmonary progenitor cells for pulmonary endothelial repair and delayed the thickening of distal pulmonary vessels	Chitosan	Intratracheal (aerosol inhalation)	Athymic nude rat, Sprague–Dawley rat	[93]

BMPR2, bone morphogenetic protein receptor type 2; PDE5, phosphodiesterase type 5; Pdgfb, platelet-derived growth factor B; PEG-PLGA, poly-(ethylene glycol)-block-lactide/glycolide copolymer; PLGA, poly(lactic-co-glycolic acid); PPAR-γ, peroxisome-proliferator-activated-receptor-gamma; PPMS, poly(pentadecalactone-co-n-methyldiethanolamineco-sebacate); SDF, stromal cell-derived factor-1α; SIRT1, silence information regulator 1; SOD, superoxide dismutase; TRPC1, transient receptor potential cation channel 1; VEGF, vascular endothelial growth factor.

## Data Availability

No new data were created or analyzed in this study. Data sharing is not applicable to this article.
